# Impact of Subacute Exposure to T-2 Toxin and Zearalenone on the Pharmacokinetics of Midazolam as CYP3A Probe Drug in a Porcine Animal Model: A Pilot Study

**DOI:** 10.3389/fphar.2019.00399

**Published:** 2019-04-16

**Authors:** Wim Schelstraete, Mathias Devreese, Siska Croubels

**Affiliations:** Laboratory of Pharmacology and Toxicology, Department of Pharmacology, Toxicology and Biochemistry, Faculty of Veterinary Medicine, Ghent University, Ghent, Belgium

**Keywords:** mycotoxins, cytochrome P450, microsomes, pharmacokinetics, drug interactions

## Abstract

Cytochrome P450 enzymes (CYP) are important catalyzing proteins involved in the biotransformation of endogenous and xenobiotic compounds. However, their expression and/or activity can be altered by exposure to contaminants such as mycotoxins. *In vitro* incubations in porcine hepatic microsomes revealed a potent inhibition of the midazolam (CYP3A) biotransformation by T-2 toxin (T-2) (Ki = 27.0 ± 3.97 μM) and zearalenone (ZEA) (Ki = 1.1 ± 0.22 μM). Consequently, the *in vivo* impact of 2 weeks exposure to T-2 (1,000 μg/kg feed) or ZEA (500 μg/kg feed) on the pharmacokinetics (PK) of midazolam (MDZ) as a CYP3A probe drug was investigated in pigs, and was compared to a control group receiving no mycotoxins. MDZ was chosen as this drug undergoes substantial first-pass metabolism in humans with equal contribution of the intestine and liver. Each pig received a single intravenous (0.036 mg/kg BW) and oral (0.15 mg/kg BW) dose of midazolam (MDZ). For the IV bolus no differences were observed in PK between control and mycotoxins exposed groups. However, oral plasma concentration-time profiles showed quantitative differences in absolute oral bioavailability *F*[*p*-value (ANOVA) = 0.022], AUC_0-inf (μg^∗^h/L) [*p*-value (ANOVA) = 0.023], Ke (1/h) [*p*-value (ANOVA) = 0.004], and Ka (1/h) [*p*-value (ANOVA) = 0.031]. Although only differences in Ke estimates after oral administration reached significance in the *post hoc* analysis due to inequality of the variances. We hypothesize that the observed trends after ZEA and T-2 exposure are related to the cytotoxic effect of T-2, resulting in an increased absorption rate constant Ka. For ZEA, an inhibition of the CYP3A enzymes is suggested based on the *in vitro* inhibition potential and increase in oral bioavailability. Further research is required to confirm the current hypothesis.

## Introduction

Cytochrome P450 enzymes (CYP) are a superfamily of drug metabolizing enzymes facilitating drug elimination (Nebert and Dalton, 2006). It is well known that xenobiotics can alter the CYP metabolism by inducing or inhibiting these enzymes either directly or through regulating mechanisms (Tanaka, 1998). Such alterations can lead to clinical relevant pharmacokinetic drug-drug and drug-food interactions. Examples are co-administration of the strong CYP3A inhibitor ketoconazole with maraviroc, of itraconazole with erythromycin, and of the intestinal CYP3A inhibiting compounds in grapefruit-juice with felodipine (Bailey et al., 1998; Tanaka, 1998; Abel et al., 2008).

Changes in drug disposition can also occur when drugs and food-contaminants are co-ingested. However, such interactions are far less investigated then the previous mentioned drug-drug and drug-food interactions. One of the most abundant food and feed contaminants are mycotoxins, contaminating up to 72% of the feed (Streit et al., 2013; Lee and Ryu, 2017). With deoxynivalenol (DON), zearalenone (ZEA), fumonisin B1 (FB1), and T-2 toxin (T-2) as the most important *Fusarium* mycotoxins in terms of prevalence and/or toxicity (Streit et al., 2013; Escrivá et al., 2015).

Deoxynivalenol and T-2 toxin exert their toxicity through inhibition of the protein synthesis and the activation of mitogen activated protein kinases, leading to inflammation, apoptosis, and altered immune responses (Pestka, 2007; Escrivá et al., 2015). The molecular targets of ZEA are estrogen receptors, causing alteration in the reproductive tract (Fink-Gremmels and Malekinejad, 2007; Streit et al., 2013; Escrivá et al., 2015). Finally, FB1, is a very potent ceramide synthase inhibitor, resulting in the accumulation of sphinganine and sphingosine ultimately causing apoptosis, and growth inhibition (Escrivá et al., 2015).

Although the mechanism of action of DON, ZEA, T-2, and FB1 are well studied, information about their impact on CYP is scant. Furthermore, the influence on the *in vivo* disposition of substrate drugs, after mycotoxin exposure, is even less subject to investigation. Up to now, the available studies focus primarily on the impact of mycotoxins on the regulation of these enzymes (Guerre et al., 2000; Spotti et al., 2000; Meissonnier et al., 2008; Ayed-Boussema et al., 2010; Duca and Mabondzo, 2010; Goossens et al., 2013; Osselaere et al., 2013; Antonissen et al., 2017).

In rats, FB1 can induce CYP2E1 activity while reducing CYP2C11 and CYP1A2 activity (Spotti et al., 2000), whereas in broiler chickens FB1 can induce CYP1A4 expression (Antonissen et al., 2017). In the latter reference also the influence of DON on the CYP1A4, CYP1A5, and CYP3A37 mRNA expression was investigated, but the results were not significant (Antonissen et al., 2017). In mice, however, DON induced CYP1A and CYP3A related activities when the dose was 0.071 mg/kg body weight (BW) and higher. Exposure to 0.25 mg/kg BW of T-2 on the other hand, reduced CYP1A1/2, CYP2A1, and CYP2B4 activity in rabbits (Guerre et al., 2000). In broiler chickens, contamination of 752 μg T-2/kg feed, was able to reduce the mRNA expression of CYP1A4/5 and CYP3A37, in contrast to an increased activity in CYP3A related reactions (Osselaere et al., 2013). In the pig, feed contaminated with 903 to 2,102 μg/kg T-2 significantly reduced CYP3A and CYP1A activities (Meissonnier et al., 2008; Goossens et al., 2013). ZEA has shown to be able to induce CYP3A4, CYP2B6, and CYP1A1 in addition to the activation of CYP regulation proteins like the pregnane X receptor (PXR), constitutive androstane receptor (CAR), and arylhydrocarbon receptor (AhR) in primary human hepatocytes (Ayed-Boussema et al., 2010). These observations were also made in PXR transfected HepG2 cells where ZEA induced the CYP3A4 expression (Ding et al., 2006). In addition, ZEA and T-2 have been proven to be, in part, metabolized by CYP enzymes in microsomes and by recombinant enzymes, which raises a risk for interactions with drugs (Bravin et al., 2009; Pfeiffer et al., 2009; Ge et al., 2010; Lin et al., 2015). However, the direct impact of these toxins on the biotransformation capabilities of CYP enzymes and *in vivo* disposition of victim substrate drugs is unknown.

The different species used in previous experiments like mouse, rat, and rabbits are often not a representative model to estimate the potential risk for humans (Bogaards et al., 2000; Zuber et al., 2002; Cao et al., 2006; Turpeinen et al., 2007). Pigs however, have been demonstrated to have a good resemblance in CYP biotransformation to human drug CYP metabolism (Millecam et al., 2018; Schelstraete et al., unpublished). Furthermore, they have a similar anatomy and physiology of the gastro-intestinal tract, liver, and circulatory system (Bode et al., 2010; Swindle et al., 2012). These features are vital for a representative animal model as in the *in vivo* setting, the intestine, and liver are the two main sites of metabolism. Thus, when investigating food contaminant effects on *in vivo* drug metabolism, substrates undergoing intestinal, and hepatic biotransformation are preferred substrate drugs. An example of such substrate is MDZ. It is almost completely absorbed and undergoes first-pass metabolism with equal contribution of the intestine and liver (Thummel et al., 1996).

Therefore the goals of the present study were twofold. The first goal was to investigate the direct effects of ZEA, T-2, DON, and FB1 on 6 major porcine CYP enzymes. These direct effects, in addition to an altered regulation of these enzymes, can lead to changes in the disposition of victim substrate drugs, eventually resulting in treatment failure, toxic responses, or altered withdrawal times. Where inhibition is revealed, the type of inhibition mechanism is elucidated. Second, these results are used as guidance to select a relevant mycotoxin-CYP3A substrate interaction and evaluate the impact on the *in vivo* disposition of MDZ as CYP3A model substrate drug in pigs during a pilot study.

## Materials and Methods

### Chemicals and Reagents

Zearalenone, T-2, DON, and FB1 were obtained from Fermentek (Jerusalem, Israel). Phenacetin (PH), paracetamol (PAR), tolbutamide (TB), 7-hydroxy-coumarin (OH-CM), dextrorphan-D3, coumarin (CM), chlorzoxazone (CZ), and trifluoroacetic acid (TFA) were delivered by Sigma-Aldrich (St. Louis, MO, United States). MDZ, 1-hydroxy-midazolam (OH-MDZ), OH-MDZ-D4, 4-hydroxy-tolbutamide (OH-TB), 6-hydroxy-chlorzoxazone (OH-CZ) were purchased at LGC standards (Molsheim, France). Dextromethorphan (DXM), dextrorphan tartrate (DEX), 7-hydroxycoumarin-D5, paracetamol-D4, and OH-TB-D9 were from Toronto Research Chemicals (North York, ON, Canada). OH-CZ-^13^C_6_ was obtained from Alsachim (Illkirch Graffenstaden, France). NADPH was obtained from OYC Europe (Rotterdam, Netherlands). All other chemicals were of HPLC grade. Potassium chloride, potassium dihydrogenphosphate, potassium hydrogenphosphate, formic acid, and glycerol were bought at VWR (Leuven, Belgium).

Stock solutions of each substrate and toxin were prepared in MeOH (MDZ, 3.26 mg/mL; CM, 0.13 mg/mL; DXM, 6.67 mg/mL; PH, 16.14 mg/mL; T-2, DON, ZEA, and FB1 1 mg/mL) or ACN (TB, 48.66 mg/mL; CZ, 15.26 mg/mL; SFZ, 0.32 mg/mL; DDC, 9.01 mg/mL) and stored at -20°C. Fresh working solutions were prepared by adding an appropriate amount of stock solution to HPLC-quality water. The stop reagent consisted of 55% ACN, 42% HPLC water, and 3% formic acid with internal standards (final concentrations: 40, 100, 100, 200, 200, and 40 ng/mL for OH-MDZ-D4, OH-CZ-^13^C_6_, OH-TB-D9, OH-CM-D5, PAR-D4, and DXT-D3, respectively). All stock solutions were stored at -20°C.

### *In vitro* Experiments

#### Animal Tissues and Preparation of the Microsomes

Sixteen livers were obtained from conventional pigs (hybrid sow × Piétrain boars, 12 weeks of age, 8 boars and 8 sows) reused from a previous trial (approval EC2015_213) in the context of animal reduction. After euthanasia with pentobarbital, with prior sedation of a Zoletil^®^ (tiletamine-zolazepam, 20 mg/mL) and Xyl-M^®^ (xylazine, 2%) mixture 1/2 (m/m), livers were excised and rinsed twice in phosphate buffered saline and immediately frozen in liquid nitrogen. Microsomes were prepared as previously described (Wilson et al., 2003). In brief, liver samples were thawed on ice, submerged in 0.25 M phosphate buffer containing 1.15% potassium chloride (pH 7.25). Subsequently, 4 grams of liver were minced and homogenized in 16 mL of a 0.25 M phosphate buffer by the use of a Potter-Elvehjem homogenizer. Samples were centrifuged for 25 min at 10,000 *g* and 4°C. After decanting, the supernatant was centrifuged for 80 min at 100,000 *g* and 4°C. Next, samples were washed with 4^∗^3 mL 0.25 M phosphate buffer and centrifuged for a second time for 80 min at 100,000 *g* and 4°C. The obtained pellet was suspended in 1.5 mL/g liver 0.25 M phosphate buffer containing 1.15% potassium chloride and 30% glycerol, snap frozen, and stored at -80°C. All handlings were performed on ice.

Microsomal proteins were determined according to the method of Bradford (Bradford, 1976), following the manufactures instructions.

#### Incubations of Microsomal CYP With and Without Mycotoxins

Each incubation was performed at 37°C in a thermoshaker TS 100 (Biosan, Riga, Latvia), and was repeated three times. Each incubation medium consisted of 50 μL of a 0.2 M phosphate buffer, 50 μL of microsomes diluted to 1.25 mg proteins/mL (for TB incubations), or 0.5 mg proteins/mL (for other incubations), 50 μL of an aqueous 1.15% potassium chloride solution, 50 μL of a substrate and/or mycotoxin working solution of different concentrations, and 50 μL of a 25 mM NADPH solution. Before adding NADPH, the mixture was pre-incubated for 5 min. The organic solvent content was kept below 1%. Incubation time was dependent on the substrate investigated and was 5 min for PH (CYP1A), CM (CYP2A), DXM (CYP2D), CZ (CYP2E), and MDZ (CYP3A). For TB (CYP2C) incubation time was 10 min. After the specified incubation times, 125 μL of stop reagent was added. Samples were further processed as described in the sample preparation and chromatographic analysis section.

To be able to identify relevant interactions, an orientation experiment was set up. In this experiment substrates were incubated with and without the addition of mycotoxins. Each mycotoxin was added to obtain a final concentration of 10 μM in the incubation mixture. To account for possible time dependent effect, the experiment was performed with and without a prior incubation of 5 min of the mycotoxins. Based on these results, mycotoxins showing inhibition (as defined by <80% residual CYP enzyme activity), as well as absolute and relative differences between the two experiments, were used as guidance for selecting mycotoxin-substrate pairs for investigation of the inhibition type. The 80% cut-off results from a simulation (*n* = 10,000) in which the addition of an inhibitor has no effect and given the precision of the analytical procedure is ≤10%. The limit was calculated such that the probability of finding a percentage lower than the threshold due to chance was ≤10%.

The selected mycotoxin-substrate pairs were incubated at varying concentrations of substrate and inhibitor as depicted in Supplementary Table 2. Incubations were identical as described above.

#### Sample Preparation and LC-MS/MS Analysis of Substrate Drug Metabolites in Incubated Microsomal Media

After adding the stop reagent, 125 μL of TFA was added and samples were subsequently centrifuged at 16,200 g for 10 min. The supernatant was decanted in 15 mL conical tubes, containing 1 mL of a 0.2 M phosphate buffer. Next, 7 mL of ethyl acetate were added and the samples were extracted during 15 min on an overhead shaker (IKA^®^ TRAYSTER, Staufen, Germany) at room temperature. The two phases were separated by centrifugation at 1,000 g for 5 min. The organic phase was transferred in a glass vial and evaporated under a gentle nitrogen stream at 40 ± 5°C. After evaporation of the organic phase, 0.2 mL of a 50/50 methanol/HPLC water mixture were added and an aliquot of 10 μL was injected into the HPLC equipment. The samples were analyzed according to a previously reported and validated HPLC-MS/MS method (Schelstraete et al., 2018).

### *In vivo* Experiments

#### *In vivo* Animal Trial With ZEA and T-2

All procedures used in the animal trial were in accordance to the ethical standards of the ethical committee of the Faculties of Veterinary Medicine and Bioscience Engineering of Ghent University (approval EC2017_91). Twenty-four conventional pigs (hybrid sow × Piétrain boars, ILVO, Melle, Belgium), 7 weeks of age, were randomly divided in 3 groups of 8 pigs (4 males and 4 females). For a 2 week period, each group was fed with either control feed, T-2 contaminated (1,000 μg/kg), or ZEA (500 μg/kg) contaminated feed. The contaminated feed was prepared by adding the toxins (analytical grade) to the compound feed. The pigs receiving mycotoxin contaminated feed were administered 0.5 kg of contaminated feed/pig in the morning and 0.5 kg of control feed/pig in the afternoon. This corresponds to a mean intake of 500 and 250 μg/kg feed of T-2 and ZEA, respectively. This correlates with a mean intake of feed contaminated with levels equal to the maximal guidance levels according to 2006/576/EG and 2013/165/EU. For the control group, 0.5 kg of control feed/pig was given at each occasion.

After the 2 weeks feeding period, MDZ was administered to each pig as a single intravenous bolus (0.036 mg/kg BW) and blood samples (±1 mL) were collected in Vacutest^®^ heparine tubes, with a 21G needle (Novolab, Geraardsbergen, Belgium) at 0, 0.083, 0.166, 0.333, 0.500, 0.666, 1, 1.5, 2, 3, 4, 6, 8, and 10 h post-administration (p.a.). Blood samples were taken by restraining the pigs and direct venipuncture of the *vena jugularis externa*. After a 48 h wash out period, the pigs were administered an oral bolus (0.15 mg/kg BW) and blood samples were collected at the same time points. The doses were selected in order to have minimal sedative effect. In line with this, the IV dose was about 1/4 compared to the PO dose. Blood samples were centrifuged for 10 min at 4,000 *g* and the supernatant plasma was transferred to a vial and stored at -20°C until analysis. At the end of the trial, pigs were euthanized as described above.

#### Determination of Midazolam and 1-Hydroxy-Midazolam in Plasma

The quantification of MDZ and OH-MDZ was performed on a Waters^®^ Alliance 2690 separation module and autosampler (Waters, Milford, CT, United States), coupled to a Waters Quattro Ultima^®^ triple quadrupole mass spectrometer (Micromass Waters, Manchester, United Kingdom). Separation was performed on a Zorbax Eclipse Plus C18 column (100 mm × 3.0 mm i.d., 3.5 μm d.p.). The mobile phases consisted of 0.1% formic acid in HPLC water (mobile phase A) and 0.1% formic acid in methanol (mobile phase B). The following gradient was used (A/B%): 0–4 min, 85:15 to 40:60; 4–4.1 min, 40:60 to 10:90; 4.1–5 min, 10:90; 5–5.1 min, 10:90 to 85:15; 5.1–10min, 85:15. The mass spectrometer features were set as previously described in Schelstraete et al. (2018). The method was in-house validated for precision, accuracy, linearity, limit of quantification (LOQ), limit of detection (LOD) specificity and carry-over following guidelines of the European Commission (2002/657/EC) and international guidelines. Limits of quantification for MDZ and OH-MDZ were 0.1 ng/mL.

### Data and Statistical Analysis

Results of the incubation experiments were analyzed using Sigmaplot^®^ version 13 (Systat Software, San Jose, CA, United States). To unravel the type of inhibition, eight different equations were fitted against the data (Supplementary Table 1). The different types were competitive, partial competitive, non-competitive, partial non-competitive, uncompetitive, partial uncompetitive, mixed, and partial mixed inhibition. Model selection was based on inspection of the residual plots, Akaike information criterion, passing homogeneity tests for residuals and significance of the parameter estimates.

The pharmacokinetic (PK) data were processed using Phoenix^®^ version 8.1 (Certara, Princeton, NJ, United States). A non-compartmental analysis (NCA) was performed to calculate the area under the plasma concentration-time curve (AUC_0-inf), clearance (Cl), volume of distribution (Vd), and the elimination rate constant (ke) after IV dosing. AUC_0-inf, Cl, Vd, Cmax (maximal plasma concentration), Tmax (time to Cmax), Ke, absorption rate constant (Ka), and F (absolute oral bioavailability) were calculated after PO dosing. Oral Cl and Vd were corrected for the bioavailability F.

Statistical analysis of the estimated parameters was performed in SPSS version 25 (IBM, Armonk, NY, United States). The parameters were log-transformed and ANOVA was carried out to detect mean differences amongst the groups. A *post hoc* Tukey test was used if the variances were equal, as evaluated by the Levene’s test. If the assumption of equal variances was violated, a Dunnett’s T3 test was performed. The significant level was set at α = 0.05.

## Results

### Orientation Experiment

The results of the orientation experiments are presented in Table 1. Deoxynivalenol inhibited none of the reactions more than 20% without pre-incubation of the mycotoxins, therefore DON was not considered for further investigation. In contrast, ZEA could strongly inhibit the CYP2C (TB-hydroxylation) and CYP3A (MDZ-hydroxylation) activities and slightly the CYP2D (DXM-demethylation) activity. Consequently they were selected for further investigation. ZEA could also markedly reduce CYP2E (CZ-hydroxylation) related activities, however, recent data show that this reaction can also be catalyzed by CYP3A, CYP2C, and CYP2A enzymes (Schelstraete et al., unpublished). Because the reduction of CZ-hydroxylation is paralleled by a reduction in CYP3A and CYP2C activities upon incubation with ZEA and by a reduction in CYP2A (CM-hydroxylation) activity upon incubation with FB1, it was hypothesized that the CYP2E enzyme was probably not inhibited by the investigated mycotoxins.

**Table 1:** Results from the orientation experiment.

	TB (CYP2C)	CZ (CYP2E)	CM (CYP2A)	PH (CYP1A)	MDZ (CYP3A)	DXM (CYP2D)
**Without mycotoxin pre-incubation**						
DON	111	92.8	87.2	95.6	102	98.0
ZEA	9.40	39.1	96.1	97.0	16.6	73.8
T-2	62.3	79.5	99.1	96.7	115	99.1
FB1	110	69.8	19.3	70.9	118	91.5
**With mycotoxin pre-incubation**						
DON	68.8	57.6	42.5	64.9	66.0	68.4
ZEA	5.50	27.9	73.4	62.7	7.50	66.3
T-2	46.3	65.4	72.5	78.2	34.4	94.2
FB1	77.5	59.2	12.6	49.5	78.8	75.5

*Results are expressed as mean residual activity compared to control incubation without mycotoxin (%, *n* = 3). Mycotoxins were added 5 min before the substrate if a pre-incubation step was included. DON, deoxynivalenol; ZEA, zearalenone; T-2, T-2 toxin; FB1, fumonisin B1; TB, tolbutamide; CZ, chlorzoxazone; CM, coumarin; PH, phenacetin; MDZ, midazolam; DXM, dextromethorphan. The full dataset can be found in Supplementary Data Sheet 1.*

T-2 toxin could moderately inhibit the CYP2C biotransformation without pre-incubation of the toxin. However, after pre-incubating T-2 for 5 min, the CYP3A activity was strongly reduced. The relative reduction in CYP3A activity (compared to the incubation without mycotoxin pre-incubation) was higher compared to the other investigated reactions. Therefore, the interaction between T-2 and MDZ was studied more profoundly after pre-incubation of the mycotoxin, while for the interaction with TB no pre-incubation was included.

Fumonisin B1 reduced the CYP2E (CZ), CYP2A (CM), and CYP1A (PH) enzyme activity by more than 20%. Given that the inhibition of CM was very strong and the fact that simultaneous experiments (Schelstraete et al., unpublished) showed that porcine CYP2A19 also contributes to PH and CZ biotransformation, the moderate inhibition of CZ and PH was considered to arise from the inhibition of CYP2A19 and was therefore not considered for further elaboration.

### Evaluation of the Inhibition Type of the Selected Mycotoxins

The results of the curve fitting analysis from incubations with different concentrations of mycotoxins and probe substrate are given in Table 2. In this table, the Vmax, and Km values are the values corresponding to an inhibitor concentration of zero. The functional relation between the parameters is shown in Supplementary Table 1, where the equations can be found. Noticeable is that ZEA and T-2 could fairly potent inhibit the MDZ and TB biotransformation, as demonstrated by relative low inhibitory constant (Ki) values. Additionally, FB1 showed a high affinity toward CYP2A19 enzymes (CM-hydroxylation) since the Ki value was low, although the efficacy (17% inhibition at highest FB1-CM concentration) was low compared to that of T-2 and ZEA toward CYP3A and CYP2C enzymes (MDZ- and TB-hydroxylation, <30% residual activity). As a consequence, ZEA, and T-2 were selected for the assessment of their impact on the *in vivo* disposition of MDZ in the pig.

**Table 2:** Results of the curve fitting analysis.

Interaction	Inhibition type	Vmax (pmol/mg protein/min)	Km (μM)	Ki (μM)	α	β	Residual activity^∗^ (%)
ZEA-TB	Partial competitive	160 (16.0)	1074 (137.9)	0.54 (0.043)	9.5 (1.05)	NA	18.7 (0.35)
ZEA-MDZ	Partial mixed	2155 (73.8)	12.3 (1.10)	1.1 (0.22)	3.8 (1.43)	0.26 (0.064)	27.0 (3.16)
ZEA-DXM	Full non-competitive	1971 (39.7)	2.0 (0.17)	55.4 (5.77)	NA	NA	32.0 (1.27)
T-2-TB	Full competitive	132 (17.9)	804 (150.0)	14.8 (1.45)	NA	NA	24.0 (1.38)
T-2-MDZ	Full non-competitive	1899 (88.2)	11.3 (1.32)	27.0 (3.97)	NA	NA	83.5 (2.41)
FB1-CM	Full mixed	359 (4.1)	0.91 (0.051)	2.3 (0.34)	14.0 (4.30)	NA	22.8 (3.50)

*Parameter estimates are expressed as mean value (standard deviation). For each interaction, 4 different mycotoxin concentrations were used in combination with 6 different substrate concentrations. Each incubation was performed in triplicate. ZEA, zearalenone; T-2, T-2 toxin; FB1, fumonisin B1; TB, tolbutamide; CM, coumarin; MDZ, midazolam; DXM, dextromethorphan; Km, Michaelis-Menten constant; Ki, inhibitory constant; α, affinity constant modulator for enzyme-inhibitor complex; β, catalytic constant of the enzyme-substrate-inhibitor complex; NA, not available. ^∗^Residual activity at the highest concentration of substrate and inhibitor. The full dataset can be found in Supplementary Data Sheet 2*.

### *In vivo* Impact of ZEA and T-2 on the Disposition of Midazolam

**Figure 1:**
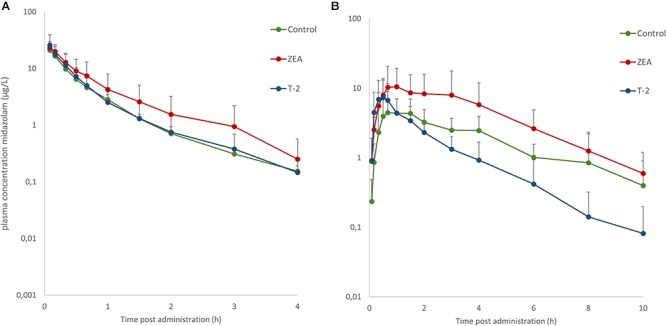
Mean (+SD) plasma concentration-time profiles for midazolam (MDZ) after administration of a single intravenous dose of 0.036 mg/kg BW **(A)** and oral dose of 0.15 mg/kg BW **(B)**. Control and T-2 group: *n* = 8, 4 boars and 4 sows, 9 weeks of age; ZEA group: *n* = 7, 4 boars and 3 sows, 9 weeks of age.

Mean plasma concentration-time profiles of MDZ after both administrations are displayed in Figure 1. Parameter estimates resulting from PK NCA after IV and PO dosing are given in Table 3.

**Table 3:** PK parameter estimates obtained by non-compartmental analysis after IV dosing of midazolam (0.036 mg/kg BW) and PO dosing of midazolam (0.15 mg/kg BW).

Administration route	Parameter	Control	ZEA	T-2
IV	AUC (0-inf) (μg^∗^h/L)	11.5 (1.51)	18.0 (12.44)	13.0 (6.69)
	Vd (L)	60.8 (23.17)	63.4 (35.02)	62.9 (32.01)
	Cl (L/h)	46.0 (7.51)	45.5 (22.75)	45.8 (16.41)
	Ke (1/h)	0.83 (0.279)	0.69 (0.176)	0.79 (0.215)
	MRT (h)	0.71 (0.110)	0.92 (0.472)	0.63 (0.086)
PO	AUC (0-inf) (μg^∗^h/L)	18.4 (7.51)	46.1 (39.9)	13.6 (4.62)
	Vd (L)	128 (40.4)	136 (93.8)	76.2 (43.25)
	Cl (L/h)	46.0 (7.51)	44.1 (27.73)	45.8 (16.41)
	Ke (1/h)	0.390 (0.137)	0.390 (0.121)	0.68 (0.227)
	Ka (1/h)	0.426 (0.289)	0.419 (0.130)	1.19 (0.904)
	F	0.36 (0.136)	0.69 (0.429)	0.27 (0.124)
	Cmax (μg/L)	5.45 (4.230)	12.0 (10.19)	8.85 (5.720)
	Tmax (h)	1.15 (0.490)	1.14 (0.900)	1.13 (1.270)
	MRT (h)	3.58 (0.884)	3.46 (0.840)	2.24 (1.352)

*Results are presented as mean (standard deviation) (control and T-2 group: *n* = 8, 4 boars and 4 sows, 9 weeks of age; ZEA group: *n* = 7, 4 boars and 3 sows, 9 weeks of age). AUC (0-inf), area under the plasma concentration-time curve from time = 0 min to infinity; Vd, volume of distribution; Cl, clearance; Ke, elimination rate constant; Ka, absorption rate constant; F, bioavailability; Cmax, maximal concentration; Tmax, time to maximal concentration; MRT, mean residence time. The full dataset can be found in Supplementary Data Sheet 3*.

After IV administration of MDZ, a trend for the AUC (0-inf) was apparent from the mean plasma concentration-time profile of MDZ in the ZEA group. However, no statistical significant differences in parameter estimates could be observed [lowest *p* = 0.42 for AUC (0-inf)]. Of notice are the results for Cl, Vd, and Ke amongst the three groups, with nearly identical estimates, which suggest equal elimination characteristics. This is also reflected in the profiles (Figure 1A), as the plasma concentration of MDZ declines in parallel amongst all groups.

In contrast to the kinetic profiles after an IV dose, the plasma concentration-time course of MDZ after oral dosing were more divergent. Indeed, as Figure 1B shows, an increasing trend in mean Cmax values were observed in the mycotoxin treated groups (*p* = 0.238). In addition, the AUC (0-inf) for MDZ in the ZEA group was about 2.5 times higher on average, which was significant in the ANOVA analysis (*p* = 0.023), but adjusting for the unequal variances (Levene’s test, *p* = 0.003), the *post hoc* analysis was not significant (lowest *p*-value: ZEA-T-2: *p* = 0.106; ZEA-Control: *p* = 0.263). Again, as with the IV dosing, a remarkable similar result in Cl is observed amongst the three groups. However, the average Vd estimate of the T-2 exposed group was somewhat lower, but not statistical significant (*p* = 0.174). Considering this similar Cl and lower Vd, the T-2 group showed a statistical significant higher Ke estimate (*p* = 0.004), highlighting a potential different elimination half-life in the T-2 group. Furthermore, the average Ka of the T-2 group was about 2.5 times higher compared to the control group. The result of the ANOVA was significant (*p* = 0.031), but also here the adjustment for the uneaqual variances (Levene’s test, *p* = 0.012) rendered the *post hoc* analysis not significant (lowest *p*-value: T-2-Control, *p* = 0.104). A change in Ka should result in a change in Tmax values. However, this was not reflected in the average Tmax for the T-2 group. The reason is that one pig showed a Tmax of 4 h, while the other Tmax values ranged between 0.16 and 1.5 h. In the control group this range was between 0.67 and 2 h. Removing the observation from the analysis resulted in a mean Tmax value of 0.714 ± 0.559 h. However, the result was not significantly different amongst the groups (*p* = 0.170). Lastly, the average F differed amongst the groups (ANOVA, *p* = 0.022), nevertheless this results was marginally non-significant (*p* = 0.093).

**Figure 2:**
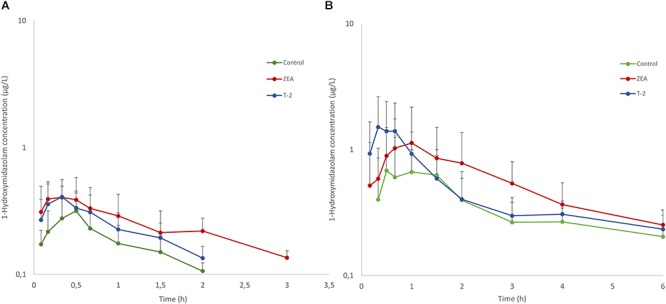
Mean (+SD) plasma concentration-time profiles for 1-hydroxymidazolam after an intravenous dose of 0.036 mg/kg BW MDZ **(A)** and an oral dose 0.15 mg/kg BW of MDZ **(B)**. Control and T-2 group: *n* = 8, 4 boars and 4 sows, 9 weeks of age; ZEA group: *n* = 7, 4 boars and 3 sows, 9 weeks of age.

Besides MDZ, also OH-MDZ -the principal MDZ metabolite- was measured and the plasma concentration-time profiles are depicted in Figure 2. Mean AUC and Cmax values can be found in Table 4. Although, by inspection of the plots in Figure 2, elimination of OH-MDZ is rather similar due to the comparable decline in plasma concentration after Cmax. The average AUC and Cmax after IV bolus administration was smaller in the control group, although not significant (*p* = 0.104). In contrast, after PO administration, AUC values for the control and T-2 group were nearly identical on average. The highest average Cmax value for OH-MDZ was observed for the T-2 and ZEA group, which aligns with the high Cmax values of the MDZ concentration in these groups (Table 4).

**Table 4:** AUC and Cmax values for OH-MDZ after IV and PO administration of midazolam.

	AUC_IV (μg^∗^h/L)	Cmax_IV (μg/L)	AUC_PO (μg^∗^h/L)	Cmax_PO (μg/L)
Control	0.46 ± 0.195	0.32 ± 0.115	2.28 ± 1.521	0.81 ± 0.587
ZEA	0.81 ± 0.557	0.48 ± 0.145	3.43 ± 1.991	1.24 ± 1.013
T-2	0.83 ± 0.642	0.41 ± 0.147	2.28 ± 0.665	1.73 ± 0.980

*Values are represented as mean ± standard deviation (*n* = 8 for control and T-2 group, 4 boars and 4 sows, 9 weeks of age; *n* = 7 for ZEA group, 4 boars and 3 sows, 9 weeks of age)*.

## Discussion

The objectives of the current study were twofold. On the one hand, the inhibitory potential and inhibition type of four frequently occurring *Fusarium* mycotoxins, DON, ZEA, T-2, and FB1 (Supplementary Figure 1), on porcine CYP enzymes were investigated. On the other hand, the influence of ZEA and T-2 on the disposition of MDZ in the pig was studied.

In the orientation experiments, DON could not inhibit any of the investigated probe reactions for CYP1A, CYP2A, CYP3A, CYP2C, CYP2D, and CYP2E. This observation aligns with previous reports, stating that DON is not subject to phase I metabolism (Côté et al., 1987; Maul et al., 2012). In contrast, T-2 could moderately inhibit the CYP2C probe reaction (TB-hydroxylation) without a pre-incubation, while relative strong inhibition was seen for the CYP3A probe reaction (MDZ-hydroxylation) after including a pre-incubation step. Wang et al. (2011) suggested a role of porcine CYP2C in the metabolism of T-2 (Wang et al., 2011). Furthermore, various members of the porcine CYP3A family have been demonstrated to convert T-2 and HT-2 toxin (HT-2) to its 3′-hydroxy metabolites (Ge et al., 2010; Wang et al., 2011; Wu et al., 2011). The current results are in agreement with these studies and indicate that porcine CYP2C and CYP3A enzymes most likely play a key role in the phase I metabolism of T-2. This is an important feature as it can possibly lead to kinetic interactions between T-2 and substrates of these enzymes.

The corresponding type of inhibition of T-2 toward TB-hydroxylation and MDZ-hydroxylation was full-competitive and full-non-competitive, respectively. The full-competitive inhibition implies that T-2 can directly inhibit CYP2C enzymes by binding to their active site, whereas the non-competitive inhibition toward CYP3A enzymes, indicates that either T-2 or a metabolite binds on an allosteric site or loss of functional enzyme occurs during incubation due to irreversible binding (Leskovac, 2004). Since the biotransformation of T-2 or HT-2 by CYP3A enzymes does not change the intrinsic reactive epoxide ring, it is unlikely that an irreversible complex is formed (Wang et al., 2011; Wu et al., 2014). However, the pre-incubation results in formation of metabolites of T-2 and HT-2 prior to incubation with substrates. Consequently, these metabolites can occupy allosteric sites, inducing conformational changes of the enzyme, rendering it less active.

The main biotransformation route for ZEA is a reduction to α-zearalenol (α-ZEL) and β-zearalenol (β-ZEL), catalyzed by steroid dehydrogenases (Malekinejad et al., 2006; Devreese et al., 2015). However, ZEA and its reduced metabolites can also be oxidized by CYP enzymes (Bravin et al., 2009; Pfeiffer et al., 2009). The CYP enzymes contributing to the oxidative metabolism of ZEA, α-ZEL, and β-ZEL are mainly the CYP1A, CYP2C, CYP3A subfamilies, next to the minor CYP2D contribution in humans (Bravin et al., 2009; Pfeiffer et al., 2009). Furthermore, in the rat the oxidations are also catalyzed by CYP2C members (Bravin et al., 2009). In the current study, ZEA could strongly inhibit the TB-hydroxylation (CYP2C) and MDZ-hydroxylation (CYP3A) reactions, and weakly the DXM-demethylation (CYP2D) reaction. Although, ZEA also inhibited CZ-hydroxylation (CYP2E). Nevertheless, this was considered to result from the inhibition of CYP3A and CYP2C enzymes, which contribute to this reaction as previously reported (Schelstraete et al., unpublished).

The strong, competitive-like inhibition of MDZ- and TB-hydroxylation suggests that porcine CYP2C and CYP3A enzymes contribute to the oxidation of ZEA and/or its metabolites. In addition, the weaker, noncompetitive inhibition of DXM-demethylation indicates a minor role of porcine CYP2D, similar as in humans (Pfeiffer et al., 2009). Therefore, the results of the inhibition pattern of ZEA and T-2 further support the appropriateness of the pig as an animal model in compound safety testing.

Hitherto no phase I metabolism is known for FB1 (Cawood et al., 1994). Although, it is known that the porcine gut microbiota can hydrolyze FB1 and that rats excrete unspecified metabolites in urine (Shephard et al., 1992; Fodor et al., 2008). In the current investigation, FB1 could reduce the CM activity at the concentrations tested in the orientation experiment. Coumarin is solely metabolized by porcine CYP2A (Schelstraete et al., unpublished). Moreover, this enzyme is the most abundant CYP enzyme in porcine liver microsomes and has a broader substrate specificity compared to human CYP2A. Therefore, it might be possible that porcine CYP2A is capable of hydrolyzing FB1 and therefore affects CM-hydroxylation. However, further research is required to strengthen this hypothesis.

From the results of the *in vitro* experiments, ZEA, and T-2 were selected for the investigation of their effects on the *in vivo* disposition of MDZ as a CYP3A probe. These toxins were selected because T-2 had a time dependent effect and ZEA can induce mRNA synthesis in human primary hepatocytes, mouse and rat livers (Ding et al., 2006; Ayed-Boussema et al., 2010; Duca and Mabondzo, 2010), hence, the effects can accumulate over time. MDZ in turn has the ideal property of being almost completely absorbed and further metabolized by both intestinal and hepatic CYP3A (Thummel et al., 1996). Consequently it allows to differentiate between the effects of the toxins on the intestine and liver.

After IV administration of MDZ to the different test groups, no differences in AUC (0-inf), Cl, Vd, and Ke were observed, indicating the effects of T-2 and ZEA on the CYP3A activity in the liver are limited. These observations are somewhat in contrast to the results of Goossens et al. (2013) were a reduced OH-MDZ formation was observed in hepatic microsomes prepared from pigs exposed to 903 μg T-2/kg feed for 14 days (Goossens et al., 2013). Possibly these discrepancies can be attributed to the difference in exposure to T-2. In this study, pigs were exposed on average to 500 μg of T-2/day vs. 903 μg/kg feed fed *ad libitum* in the mentioned study. Furthermore, when T-2 is administered orally to pigs, even at doses of 2.4 mg/kg BW, no parent toxin could be detected in plasma nor liver (Beasley et al., 1986), indicating that T-2 is unlikely to directly affect the metabolism in the liver due to the high intestinal biotransformation (Beasley et al., 1986; Wu et al., 2012). In addition, after intra-aortal administration of 1.2 mg T-2 to pigs, a mean elimination half-life of 13.8 min was observed, further supporting the hypothesis that T-2 is not likely to directly affect the hepatic metabolism due to high first-pass and/or short exposure time (Beasley et al., 1986).

In contrast to T-2, ZEA can induce mRNA synthesis and CYP3A activity in human primary hepatocytes, mouse, and rat hepatocytes (Ding et al., 2006; Ayed-Boussema et al., 2010; Duca and Mabondzo, 2010). However, no increased elimination of MDZ was observed *in vivo* in the current study. Again, these differences can be attributed to a different exposure to the toxin. In the *in vitro* settings, the concentrations ranged from 0.03183 to 15.92 μg/mL (Ding et al., 2006; Ayed-Boussema et al., 2010), whereas in the *in vivo* investigation, rats were exposed to 25 mg/kg BW (Duca and Mabondzo, 2010), which are high compared to a total daily intake of 250 μg ZEA in the present study.

Compared to the IV profiles, PK profiles after PO administration of MDZ were more divergent. Quantitative differences in absolute oral bioavailability *F*[*p*-value (ANOVA) = 0.022], AUC_0-inf (μg^∗^h/L) [*p*-value (ANOVA) = 0.023], Ke (1/h) [*p*-value (ANOVA) = 0.004], and Ka (1/h) [*p*-value (ANOVA) = 0.031] were observed, although only the Ke showed a significant difference (*p*-value = 0.017) after *post hoc* testing due to unequal variances observed amongst the groups. This increased variability can originate form differences in feed intake due to hierarchy within each pen, or differences in response to mycotoxin exposure. Nevertheless, some relevant trends could be noticed. Compared to the control group, pigs receiving T-2 tend to have higher average Cmax, Ke, and Ka values but a similar AUC (0-inf) estimate. The ZEA group on the other hand, showed an increasing trend in Cmax, F, and AUC (0-inf) values but with similar Ke and Ka estimates. Higher average Cmax, Ka, and F values for MDZ in the toxin treated groups indicate that MDZ is absorbed faster or less eliminated before reaching the systemic circulation. As both the intestine and the liver contribute to the first-pass metabolism of MDZ (Thummel et al., 1996), and no differences in elimination were found after IV administration, the observed differences most likely originate from changes occurring in the intestine. From the results presented in the current study and information available in other reports, it ishypothesized that this is either due to altered epithelial integrity or reduced biotransformation. However, a larger scale study is required to confirm the current findings.

There is some evidence in literature which align with this hypothesis of reduced epithelial integrity and to the observations for the PK of MDZ in the T-2 group. Indeed, T-2 is strongly cytotoxic and has the potential to interfere with the intestinal epithelial cells (Goossens et al., 2012a,b). Concentrations as low as 10 ng/mL (21.43 nM) of T-2 have been shown to significantly affect the epithelial integrity of IPEC-J2 cells causing a threefold increase in doxycycline and more than 50-fold increase in paromomycin passage across IPEC-J2 cells (Goossens et al., 2012a). With a mean intake of 500 μg T-2/day, concentrations of more than 21.43 nM (10 ng/mL) in the intestinal lumen are reasonable to assume. This increase in epithelial transfer is reflected in the higher average Ka for MDZ in the T-2 group, which was significant different between groups (ANOVA, *p* = 0.031; *post hoc* T-2-Control *p* = 0.104). As a consequence of faster absorption, Cmax values are also elevated but the AUC (0-inf) is unaffected, as the Ka has no influence on this parameter. A similar observation was made for doxycycline when pigs were exposed for 7 days to 100 μg T-2/kg feed (Goossens et al., 2012b).

Besides the Ka and Cmax, also the Ke estimate (*p* = 0.017) increased. Closer inspection of the pharmacokinetic profiles of the T-2 group vs. the ZEA and control group, reveal a double absorption peak in the latter two groups. Due to the high reactivity of T-2, it is likely that only the first part of the small intestine is affected, leading to increased or even total absorption over this initial part and consequently the absence of a second absorption peak. This faster absorption and absence of a second peak can explain the apparently higher-elimination in the T-2 group. The latter hypothesis corresponds with the observation that maximal OH-MDZ concentrations tend to be higher in the T-2 treated group.

In contrast to T-2, ZEA is less cytotoxic for the intestinal epithelial cells, only affecting IPEC-J2 cell viability at concentrations of 40 μM (12.73 μg/mL) (Wan et al., 2013). As the mean ZEA intake was only 250 μg/day, concentrations of 40 μM in the intestinal lumen are unlikely. Consequently the impairment of the intestinal barrier function is unlikely. However, for the direct inhibition of ZEA toward CYP3A enzymes, the Ki value is 1.1 μM. Assuming a combined water content of about 720 mL in the stomach and small intestine from pigs provided water *ad libitum* (Merchant et al., 2011), concentrations of 1.1 μM ZEA can be reached. In addition, ZEA is subject to entero-hepatic circulation, leading to prolonged exposure of the enterocytes to ZEA and metabolites over the length of the small intestine (Dänicke et al., 2005). Therefore it can be hypothesized that the increasing trend of Cmax, AUC (0-inf), and F in the ZEA group results from the intestinal CYP3A inhibition. Although the *in vitro* experiments were based on hepatic microsomes, we previously showed that the same CYP3A isoenzymes are expressed in the intestine (Schelstraete et al., unpublished). An inhibition of intestinal CYP3A metabolism does not influence Ka but it can have pronounced effects on F -and consequently the AUC (0-inf)- because it depends on the intestinal and hepatic first-pass. Furthermore the concentration time course for the control and ZEA groups was similar, which further supports the hypothesis of CYP3A inhibition by ZEA (or metabolites) over the length of the small intestine. Finally, the higher average Cmax for MDZ in the toxin treated groups translates in a higher average Cmax in the OH-MDZ.

Determination of the CYP3A enzymes in the intestine would have aided to confirm our hypothesis. However, the variability in activity is large and expression is different along the tract (Van Peer et al., 2014; Schelstraete et al., 2018). In addition, these enzymes were previously quantified in the intestinal microsomes (Schelstraete et al., unpublished). The CYP enzymes detected belonged to the CYP3A and CYP2C subfamily, with a total amount detected of 3.44 ± 2.42 pmol/mg protein. This value is about 100-fold lower than in hepatic microsomes. Furthermore, the detected amounts of intestinal CYP enzymes were near the analytical limits, making it extremely difficult to detect a treatment difference. Nevertheless, with the fast increase in available mass spectrometry methods and increasing sensitivity, this approach should be included in future research.

In conclusion, ZEA and T-2 have a tendency to influence the pharmacokinetics (PK) of MDZ, a typical CYP3A substrate, at realistic levels of mycotoxin contamination, although the results were only significant for Ke and marginally non-significant for F, and Ka. However, a larger follow-up study should be performed to confirm the current findings. The results of the present study allow to calculate an appropriate sample size for this future research. As T-2 and ZEA are frequently occurring in food and feed, they can affect pharmacotherapy. Indeed, alterations in biotransformation capacities can lead to an altered exposure and have deleterious effects on therapeutic outcome of substrate drugs. In addition these alterations can potentially lead to toxic responses and longer than expected withdrawal times for some therapeutics. Finally, as porcine and human CYP3A closely resemble each other, co-ingestion of ZEA and T-2 contaminated food with drugs can possibly affect human drug disposition as well.

## Ethics Statement

All procedures performed in the animal trial were in accordance to the ethical standards of the ethical committee of the Faculties of Veterinary Medicine and Bioscience Engineering of Ghent University (Approval No. EC2017_91).

## Author Contributions

All authors wrote the manuscript. WS studied the design and performed the data analysis.

## Conflict of Interest Statement

The authors declare that the research was conducted in the absence of any commercial or financial relationships that could be construed as a potential conflict of interest.
