# Supplementary material for: Impact of Subacute Exposure to T-2 Toxin and Zearalenone on the Pharmacokinetics of Midazolam as CYP3A Probe Drug in a Porcine Animal Model: A Pilot Study
Source: Front Pharmacol. 2019 Apr 16;10:399. doi: 10.3389/fphar.2019.00399 (PMC6492070; doi:10.3389/fphar.2019.00399)
Supplement: Supplementary file 4 [file Data_Sheet_4.docx]

Supplementary Table 1 Mathematical expressions for the considered inhibition types and their description.

| **Inhibition type** | **Equation** | **Description** |
| --- | --- | --- |
| **Competitive** | $V_{0}=\frac{V_{max}*\left[ S \right]}{K_{m}(1+\frac{I}{K_{i}})+[S]}$ | Inhibitor binds on the same active site as substrate |
| **Partial competitive** | $V_{0}=\frac{V_{max}*[S]}{K_{m}\frac{1+\frac{I}{K_{i}}}{1+\frac{I}{{\alpha K}_{i}}}+[S]}$ | Competitive inhibition with residual activity and different affinity for the substrate towards the enzyme and enzyme-inhibitor complex |
| **Non-competitive** | $V_{0}=\frac{V_{max}*\left[ S \right]}{\left( K_{m}+\left[ S \right] \right)*(1+\frac{I}{K_{i}})}$ | Inhibitor binds on an allosteric site of the enzyme. |
| **Partial non-Competitive** | $V_{0}=\frac{V_{max}*\left[ S \right]*(1+\frac{\beta I}{K_{i}})}{\left( K_{m}+\left[ S \right] \right)*(1+\frac{I}{K_{i}})}$ | Non-competitive inhibition with residual activity but equal affinity of the substrate towards the enzyme and enzyme-inhibitor complex |
| **Uncompetitive** | $V_{0}=\frac{V_{max}*\left[ S \right]}{K_{m}+[S](1+\frac{I}{K_{I}})}$ | Inhibitor binds only to the enzyme-substrate complex |
| **Partial uncompetitive** | $V_{0}=\frac{V_{max}*[S]}{K_{m}+[S]\frac{1+\frac{I}{{\alpha K}_{i}}}{1+\frac{I}{K_{i}}}}$ | Uncompetitive inhibition with residual activity and affinity for the substrate towards the enzyme-inhibitor complex |
| **Mixed** | $V_{0}=\frac{V_{max}*\left[ S \right]}{K_{m}(1+\frac{I}{K_{i}})+[S](1+\frac{I}{\alpha K_{I}})}$ | Inhibitor has different affinity to the enzyme and enzyme-substrate complex but without residual activity. |
| **Partial Mixed** | $V_{0}=\frac{V_{max}*\left[ S \right]*(1+\frac{\beta I}{{\alpha K}_{i}})}{K_{m}(1+\frac{I}{K_{i}})+[S](1+\frac{I}{\alpha K_{I}})}$ | Inhibitor has different affinity to the enzyme and enzyme-substrate complex with residual activity. |

V_0_, biotransformation rate; V_max_, maximal biotranformation rate; K_m_, Michaelis-Menten constant; Ki, inhibition constant; [S], substrate concentration; α affinity constant modulator for enzyme-inhibitor complex; β catalytic rate constant modulator for enzyme-inhibitor-substrate complex

Supplementary Table 2 Used substrate and mycotoxin concentrations for the investigation of inhibition type in porcine hepatic microsomes. Only substrates (corresponding to a specific CYP enzyme) for which residual CYP enzyme activity is <80% in the orientation experiment were selected

| Substrate concentrations (µM) | | | | | | |
| --- | --- | --- | --- | --- | --- | --- |
| TB (CYP2C) | 5 | 10 | 50 | 100 | 200 | 400 |
| DXM (CYP2D) | 0.1 | 0.5 | 1 | 5 | 20 | 100 |
| MDZ (CYP3A) | 0.5 | 2 | 5 | 10 | 20 | 50 |
| CM (CYP2A) | 0.25 | 0.5 | 2 | 5 | 20 | 100 |
| Mycotoxin concentrations for each substrate (µM) | | | | | | |
| ZEA-DXM | 0 | 1 | 10 | 100 | | |
| ZEA-TB | 0 | 0.1 | 1 | 10 | | |
| ZEA-MDZ | 0 | 0.5 | 5 | 20 | | |
| T-2-TB | 0 | 1 | 10 | 50 | | |
| T-2-MDZ | 0 | 1 | 10 | 100 | | |
| FB1-CM | 0 | 0.1 | 1 | 5 | | |

ZEA, zearalenone; T-2, T-2 toxin; FB1, fumonisin B1; TB, tolbutamide; CM, coumarin; MDZ, midazolam; DXM, dextromethorphan

| 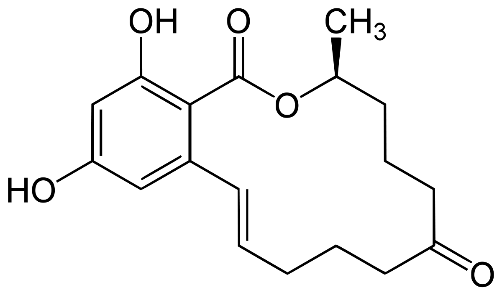 | 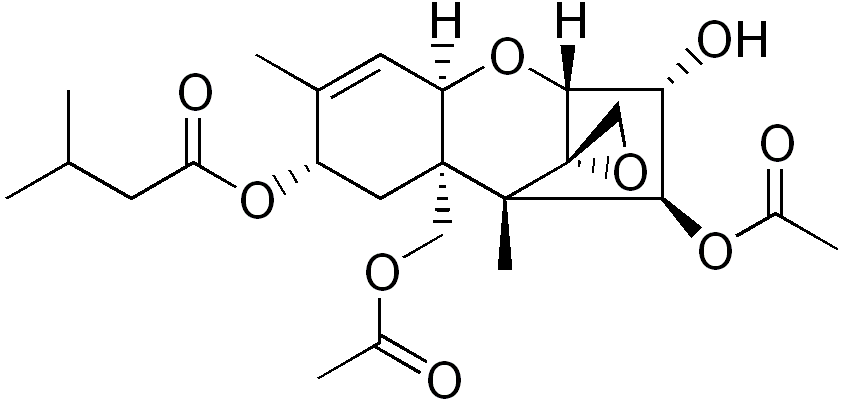 |
| --- | --- |
| 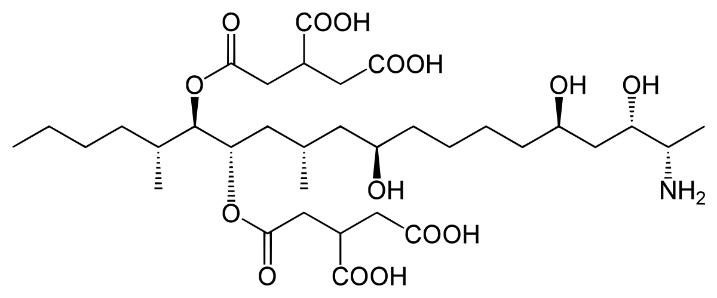 | 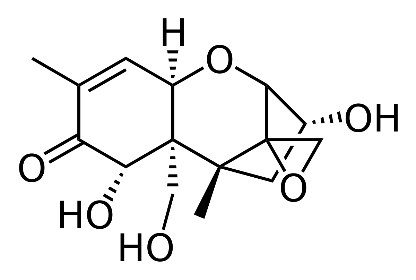 |
| Supplemental Figure 1. Chemical structures of the investigated mycotoxins. Upper left, zearalenone; upper right T-2 toxin; lower left, fumonisin b1; lower right, deoxynivalenol | |
